# Incidence of malignancies after lung transplantation and their effect on the outcome. 26 years' experience

**DOI:** 10.1016/j.heliyon.2023.e20592

**Published:** 2023-09-30

**Authors:** Konstantina Spetsotaki, Achim Koch, Christian Taube, Dirk Theegarten, Markus Kamler, Nikolaus Pizanis

**Affiliations:** a Department of Thoracic Transplantation and Assist Devices, Cardiothoracic Surgery, West German Heart and Vascular Center, University Hospital Essen, Germany; b Department of Pneumology, Ruhrland Clinic, University Hospital Essen, Germany; c Institute of Pathology, University Hospital Essen, Germany

**Keywords:** Lung transplantation, Malignancies, Lung recipient, Lung transplant, Patient survival

## Abstract

**Background:**

Malignancy is a significant, life-limiting complication after lung transplantation (LuTx) and the second common long-term cause of death. We aimed to investigate its incidence and effect on the outcome.

**Methods:**

This is a retrospective observational study. Between 1996 and 2022, n = 627 lung transplantations (LuTx) were performed in our department. We used our institutional database to identify recipients with malignancies after LuTx and examined the malignancies’ incidence and mortality.

**Results:**

N = 59 malignancies occurred in n = 55 (8.8%) LuTx recipients. The post-LTx malignancies incidence was 9.4% (59/627). We report the following rates based on their location: n = 17/55 (28,8% of all recipients diagnosed with malignancies) skin, n = 10/55 (16,95%) gastrointestinal, n = 9/55 (15,3%) respiratory, n = 5/55 (8,48%) lymphatic, n = 13/55 (23,6%) other, n = 5 (8,48%) multiple synchronous.

During this study period, a total of n = 328 deaths after LuTx was determined. N = 29 (8,84% of all deaths) were malignancy induced, corresponding to a total malignancy-induced mortality of 4.6% (n = 29/627). The majority of deaths were attributed to GI adenocarcinoma and PTLD. Malignancies’ origin, primary COPD diagnosis, type, and specific age group were significantly survival-related (p-values <0.05). The most affected organ was skin and showed the best prognosis. PTLD had the fastest and pancreatic the latest onset.

**Conclusions:**

This is the first report of its kind in a large cohort of german LuTx recipients. The prevalence ranking of the three commonest malignancy were skin > colorectal > PTLD. Post-LTx malignancy was the second commonest cause of death. Further studies are needed, while post-LuTx malignomas remain a serious impairment of long-term LuTx survival.

## Abbreviations

**Adenoca adenocarcinoma**
A1AT: α-1 antitrypsin deficiency
BCC: basal cell carcinoma
CLAD: chronic lung allograft dysfunction
CNI: calcineurin inhibitors
COPD: chronic obstructive pulmonary disease
CF: cystic fibrosis
DLuTx: double lung transplantation
EBV: Epstein-Barr virus
HCC: hepatocellular carcinoma
HPV: Human papilloma-virus
IPF: idiopathic pulmonary fibrosis
GI: gastrointestinal
LF: lung fibrosis
LuTx: lung transplantation
MCC: Merkel cell carcinoma
MD: malignant disease
MID: Malignancy induced deaths
MOD: Multiple organ dysfunction
PTLD: Post transplantation lymphoproliferative disorder
RCC: renal cell carcinoma
Re: DLuTx redo double lung transplantation
Re SLuTx: redo single lung transplantation
SCC: squamous cell carcinoma skin
SCLC: Small-cell lung cancer
SLuTx: single lung transplantation
TGF-β: transforming growth factor-beta

## 1 Introduction

Lung transplantation (LuTx) is an established treatment for selected patients resulting in significant improvement of survival and quality of life in the modern era. According to the International Society for Heart and Lung Transplantation (ISHLT), lung recipients show a median survival of 8.7 years [1]. However, malignancy is a significant, life-limiting long-term complication after LuTx, the third cause of death during the first post-LuTx year [2] and the second most common in long-term [3,4]. According to the ISHLT registry, 5% of recipients who survive 5 years after LuTx are diagnosed with any solid organ malignancy other than skin or lymphoma [5]. Cancer risk in LuTx recipients is 4.28-fold higher than in the general population, due to a variety of malignancy-inducing factors [6]. Some of those are immunosuppression and other therapies that lessen the natural anti-tumor protective mechanisms, oncogenic viruses such as EBV, underlying lung conditions per se, such as chronic obstructive pulmonary disease (COPD) and lung fibrosis (LF), sun exposure, smoking, while even the kind of single LuTx (SLuTx) relates to higher rates of new-onset native lung cancer [[7], [8], [9], [10], [11], [12]]. More specifically, the underlying oncogenic pathways for tumorigenesis after LuTx are complex and involve environmental, cellular, molecular, and genetic factors [13]. Moreover, these are related to the “double-edged sword” of immunosuppressive therapies, due to the known oncogenic effect of calcineurins inhibitors such as cyclosporine and tacrolimus. Some of these oncogenic mechanisms include DNA repair disruption, microsatellite DNA instability, the inhibition of damaged cell apoptosis, upregulation of angiogenic growth factors and tumor growth factor-β (TGF-β), the activation of oncogenic viruses that contribute to carcinogenesis, tumor progression, and metastasis [[13], [14], [15], [16]]. Several post-LuTx malignancies can occur, with their prevalence and rankings differing significantly from those of the general population. According to some studies, the systems in highest risk post-LuTx are lymphatic, hematological, integumentary, respiratory, and digestive [8,17,18]. Shen et al. report, in a 10-year review of the literature, a 10.1% incidence of malignancy after LuTx [19]. Furthermore, Fan Ge et al. report in an analysis of 21 prospective cohorts, that the most common five systems with the highest malignancy occurrence after LuTx were lymphatic and hematological systems > skin > respiratory > digestive > reproductive and urinary systems [6].

Prognosis of the most malignancies in LuTx recipients is poor, due to their aggressive malignant behavior, consequently resulting in dramatic impairment of post-LuTx long-term survival. According to ISHLT, the 5–10 post-LuTx years malignancy induced death (MID) rate is 17,3% [1]. While, MID rates of about 4,2% (6th cause of death) in the first, 12,7% (4th cause of death) in the 1st-5th and 16,3% (second cause of death) after the 5th post-LuTx year are reported [11]. It was the aim of our study to report the incidence and mortality of malignancies after LuTx based on our 26 years’ experience.

## 2 Methods

### 2.1 Data source and cohort

This is a single-center retrospective study. All the patients who received a LuTx at our department between January 1996 and August 2022 were included. Recipients who developed malignancy were confirmed. Clinical data of those were examined using the database of our institution. The origin and type of malignancy, time interval between LuTx and malignancy onset, age at the time of malignancy, overall survival, survival after malignancy onset, mortality rates, and the association of those were evaluated. Malignancies were ascertained based on the International Classification of Diseases for Oncology [20]. We defined survival as the time from lung transplantation to the day of death or loss of follow-up, up to August 31, 2022. The local ethical board (Ethic commission Duisburg-Essen University, IRBP 22-11052-BO) approved the study. Due to the retrospective nature of our study, our institutional ethical board waived informed consent. The general post-transplant approach was based on the department's protocol and the multidisciplinary approach of lung-transplantation experts, and individualized for each recipient. A triple combination of drug regimen Immunosuppression (mostly composed of tacrolimus/mycophenolate mofetil/Prednison) and ATG induction-therapie was applied in our center. All patients were managed, receiving individualized maintenance therapie in a multidisiplinary approach. All patients received post-LuTx critical care monitoring, post-LuTx complications controls and screening in the department of pneumologie, with post-LuTx follow-up appointments at least every 3 months during the first post-LuTx year and at least twice a year after the first post-LuTx year. In case of post-LuTx malignancy, a multidisiplinary approach through transplant team and oncological board was applied, an individualized reduction and adjustment of the immunosuppression, such as the use of Sirolimus-based immunosupression, as well as a multidisciplinary “case-by-cases” tumor treatment strategy consensus was applied.

### 2.2 Statistical analysis

The Shapiro-Wilk test was applied for all numerical variables to examine the normality distribution. For data that were not normally distributed, median with inter-quartile range (IQR) was used. Mean ± standard deviation was used for variables with normal distribution. The comparison between groups was performed using Mann Whitney *U* Test. All categorical variables were presented as frequencies and percentages (N%). For the comparison of categorical variables X2 (X^2^) test was applied. Survival analysis Kaplan Meier was performed in order to examine whether there was significant difference between the survival time for each malignancy category - based on malignancy origin and type respectively. Additionally, we examined how primary diagnosis, gender, age at the time of LuTx, and age at the time of malignancy onset were associate with overall, post-LuTx, and post-malignancy survival. Finally, Analysis of Variance (ANOVA) was used to examine the difference between groups for each numerical variable. P-values <0.05 are considered statistically significant. All statistical analyses were implemented using IBM SPSS Statistics - Version 26.0 (SPSS Inc., Chicago, IN, USA).

## 3 Results

### 3.1 Clinical characteristics

During the study period, n = 627 LuTx were performed. Of these, 603 DLuTx, 20 ReDLuTx, 3 SLuTx, and 1 ReSLuTx. A total of 55 recipients were diagnosed with malignancies. Of those, n = 4/55 were diagnosed with more than one synchronous primary malignancy, resulting in a total of 59 malignancies. 33/55 (60%) were males, with an average age at LuTx of 53 (range 22–66) years. Hence, the majority of n = 32 (58.2%) recipients were in the age-group of 50–64 years at the time of LuTx. Primary diagnosis was COPD in n = 25/55 (45.5%), LF in n = 18/55 (32.7%). All recipients were free of malignancy at least 5 years prior LuTx. The detailed characteristics are presented in Table 1. Average post-transplant survival was 83 (range 18–228) months. Mean age at the time of malignancy diagnosis was 59.8 (range 24–70) years.

**Table 1:** Demographic and clinical Characteristics.

Characteristics	n (%)/average (range)
Age range at the time of LuTx, (years)	n = 55/53 (22–66)
0–17	0 (0)
18–34	2 (3.6)
35–49	15 (27.3)
50–64	32 (58.2)
≥65	6 (10.9)
Gender	
Female	25 (40)
Male	33 (60)
Underlying primary lung disorder	
COPD/A1AT	25 (45.5)
IPF	13 (23.6)
CF	4 (7.3)
Fibrotic lung disease	4 (7.3)
Exogenous allergic alveolitis	3 (5.5)
Sarcoidosis	1 (1.8)
CPFE	1 (1.8)
Bronchiolitis obliterans	2 (3.6)
Other lung diseases	2 (3.6)
Lung Transplant surgery	
DLuTx	53 (96.4)
ReSLuTx	1 (1.8)
ReDLuTx	1 (1.8)
Age at Malignancy diagnosis in years	n = 55*/58 (22–72)

LuTx lung transplantation, A1AT α-1 antitrypsin deficiency, CF cystic fibrosis, COPD Chronic obstructive pulmonary disease, IPF idiopathic pulmonary fibrosis, DLuTx double lung transplantation, ReSLuTx single lung re-transplantation, ReDLuTx double lung re-transplantation.

### 3.2 Malignancy incidence based on origin and type

N = 51/55 (92.7%) of all cases developed a single primary, while n = 4/55 (7.3%) developed more than one synchronous malignancies. In detail, we determined the following, based on origin and type: 50/59 single primary; 17 skin, 33 non-skin (9 respiratory, 5 PTLD, 10 gastrointestinal tract (8 colorectal, 2 stomach), 1 liver, 1 pancreas, 2 prostate, 3 breast, 1 renal, 1 CNS), and 9/59 were multiple synchronous malignancies that occurred in 4 recipients. Details are presented in Table 1, Table 2, Table 3.

**Table 2:** Incidence of Malignant diseases (MD).

Total of LuTx recipients	n = 627	
Recipients who developed MD	n = 55 (8.8%)	
Total of MD*	n = 59 (9.4%)	
Recipients with single primary MD	n = 50 (8%)	
Total of Recipients with multiple concomitant primary MD	n = 4 (0.64%)	
Origin of MD	Number of MD, (% of total MD)	% in Cohort
Single primary Skin	17 (28.8)	2.70
Squamous cell carcinoma (SCC)	5 (8.5)	0.80
Basal cell carcinoma (BCC)	10 (17)	1.60
Melanoma	1 (1.7)	0.16
Sarcoma	1 (1.7)	0.16
Single primary Non Skin (solid + lymphatic)	33 (56)	5.90
Single primary Solid Organ	28 (47.5)	4.63
Respiratory	9 (15.3)	1.44
Pharynx	4 (6.8)	0.64
Lung	4 (6.8)	0.64
Adenocarcinoma of the lung	1 (1.7)	0.18
Small-cell lung carcinoma (SCLC)	1 (1.7)	0.18
Unknown type of bronchial carcinoma #	2 (3.4)	0.32
Trachea +	1 (1.7)	0.16
CNS Glioblastoma	1 (1.7)	0.16
Gastrointestinal tract	10 (17)	1.60
Stomach	2 (3.4)	0.32
Colon-rectal	8 (13.6)	1.28
Other solid	8 (13.6)	1.44
Liver	1 (1.7)	0.16
Pancreas	1 (1.7)	0.16
Renal	1 (1.7)	0.16
Breast	3 (5.1)	0.48
Prostate	2 (3.4)	0.48
Single primary Lymphatic/PTLD	5 (8.5)	0.80
Hypo-cellular Myelodysplastic syndrome	1 (1.7)	0.16
B-Cell non-Hodgkin Lymphoma	1 (1.7)	0.16
Post-transplant Lymphoma	1 (1.7)	0.16
Multiple Myeloma	1 (1.7)	0.16
Acute myeloid leukemia AML	1 (1.7)	0.16
Multiple primary concomitant MD	9 (15.3)	1.44
Two synchronous primary Skin (SCC, MCC)	1 (1.7)	0.16
One skin, one solid (BCC, Prostate carcinoma)	1 (1.7)	0.16
Two Synchronous primary solid (Pharyngeal and esophageal)	1 (1.7)	0.16
Three synchronous primary; SCC of skin, esophageal adenocarcinoma and prostate carcinoma	1 (1.7)	0.16

MCC: Merkel cell carcinoma skin.

*3 recipients were diagnosed with more than one malignancies.

*# Data histological type missing.*

+ Small cell lung cancer (SCLC) diagnosed in the explanted lung of the recipient.

**Table 3:** Malignancy types Incidence & Mortality rates.

Type of malignant disease	N	Incidence % in n = 55	Mortality in the entire cohort n (%)	Mortality rate of each malignancy %	Rate of mortality among all MID %
GI adenocarcinoma	10	18.2	9 (16.4)	90	29
BCC	10	18.2	1 (1.8)	10	3.3
SCC skin	5	9.1	2 (3.6)	33	6.5
PTLD	5	9.1	4(7.3)	80	12.9
Pharyngeal SCC	4	7.3	2 (3.6)	50	3.3
Breast carcinoma *	3	5.5	1 (1.8)	33	3.3
Prostate adenocarcinoma	2	3.6	1 (1.8)	33	3.3
Other bronchial carcinoma*	2	3.6	2 (3.6)	100	6.5
Lung adenocarcinoma	1	1.8	1 (1.8)	100	3.3
HCC	1	1.8	1 (1.8)	100	3.3
Pancreatic carcinoma	1	1.8	1 (1.8)	100	3.3
RCC	1	1.8	0 (0.0)	0.0	0.0
Malignant melanoma	1	1.8	0 (0.0)	0.0	0.0
Tracheal SCC	1	1.8	0 (0.0)	0.0	0.0
SCLC	1	1.8	1 (1.8)	0.0	3.3
Glioblastoma	1	1.8	1 (1.8)	100	3.3
Sarcoma	1	1.8	1 (1.8)	100	3.3
SCC skin + MCC skin	1	1.8	1 (1.8)	100	3.3
SCC + Prostate adenocarcinoma	1	1.8	0 (0.0)	0.0	0.0
Pharyngeal SCC + esophageal carcinoma	1	1.8	1 (1.8)	100	3.3
SCC skin + Prostate adenocarcinoma + GI adenocarcinoma	1	1.8	1 (1.8)	100	3.3
Chi Square Type of Malignancy – Death (20.55)	**32.706**		**p-value: 0.04**	**p-value: 0.001**	**p-value: 0.000**

BCC basal cell carcinoma, GI gastrointestinal, HCC hepatocellular, RCC renal cell carcinoma, SCC squamous cell carcinoma skin, SCLC small lung cell carcinoma, MCC: merkel cell carcinoma skin *exact histological type unknown, data missing.

### 3.3 Malignancy onset

The mean time from LuTx to malignancy onset was 61 (6–196) months for all (n = 55) recipients and 52 months for those (n = 29) who died of malignancy. The time interval from LuTx to malignancies’ onset differed significantly and was; 8.50 ± 2.12 months for PTLD, 18 for breast, 28 for CNS, 39 ± 46.66 for pharyngeal, 42.50 ± 30.87 for skin, 45 for genital, 53 ± 60.81 for lung, 59 for liver, 60.50 ± 2.12 for stomach, 120.14 ± 51.10 for colon, and 180 months for pancreas malignancies, (p-value: 0.000), as seen in Table 5.

Malignancies’ occurrence rates per post-LuTx year were; n = 9 (15.3%) in 1st, n = 6 (10.2%) in 2nd, n = 8 (13.5%) in 3rd, n = 5 (8.5%) in 4th, n = 6 (10.2%) in 5th, n = 2 in 6th, n = 2 (3.4%) in 7th, n = 3 (5%) in 8th, n = 4 (6.8%) in 9th, n = 4(6.8%) in 10th, n = 1 (1.7%) in 11th, n = 1 in 12th, n = 1(1.7%) in 14th, and n = 4 (6.8%) in 15th post-LuTx year respectively.

60% of all malignancies occurred within the first 6 years after LuTx, while 70% by the 8th post-LuTx year. Interestingly, 25% of colorectal, 100% of pancreatic, and 100% of renal malignancies occurred late in follow-up. Those with the shorter elapse time were of lymphatic origin. As seen in Kaplan Meier survival analysis (Fig. 3, Fig. 4), in most cases, the earlier the onset of the malignancy, the lower the survival (categorical-location analysis/p-value = 0.012, malignancy type analysis/p-value = 0.004).

### 3.4 Clinical outcome

During the study's interval, n = 328 of all n = 627 recipients died. Specifically, of the n = 55 recipients with malignancies, n = 31/55 (56.4%) died (8.9% of all deaths) during the study. In n = 29/31 of those, malignancy was the leading cause of death, corresponding to 94% of all deaths of recipients with malignancies, and a MID of 4.6% (n = 29/627), 8.84% (n = 29/328) of all deaths in cohort, while n = 2/31 (6.5%) of all recipients with malignancy, died of a non-malignancy cause. N = 24/55 (43.6%) of LuTx complicated with malignomas are still alive at the time of the study. In our report, post-LuTx malignancy was the second commonest post-LuTx cause of death.

By the first post-LuTx year, malignancy caused 8/29 deaths (27.6% of MID among malignancy recipients, 2.5% of all deaths, 1.3% of MID in cohort). By the 5th post-LuTx year, malignancy caused n = 20/29 deaths (6.1% of all deaths, 3,2% of cohorts’ recipients). By the 10th year post-LuTx, n = 24/29 deaths (82.8% of MID, 7.4% of all deaths, 3.83% of all recipients) were recorded.

**Fig. 1:**
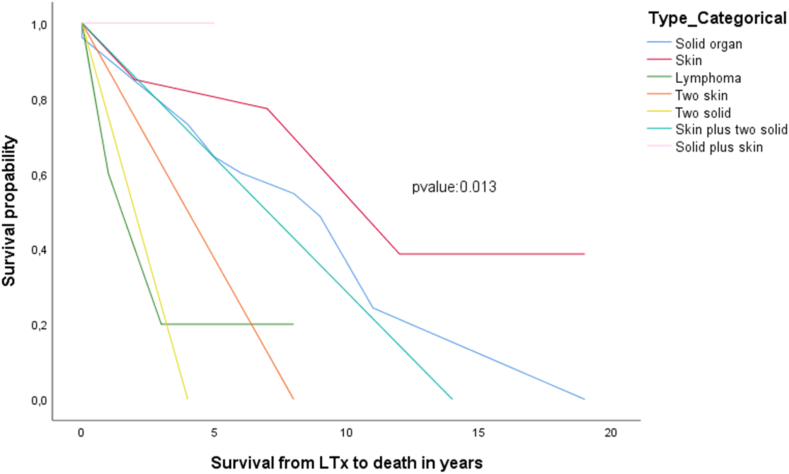
Kaplan-Meier survival by 20 years post-LuTx by location of the malignancy.

**Fig. 2:**
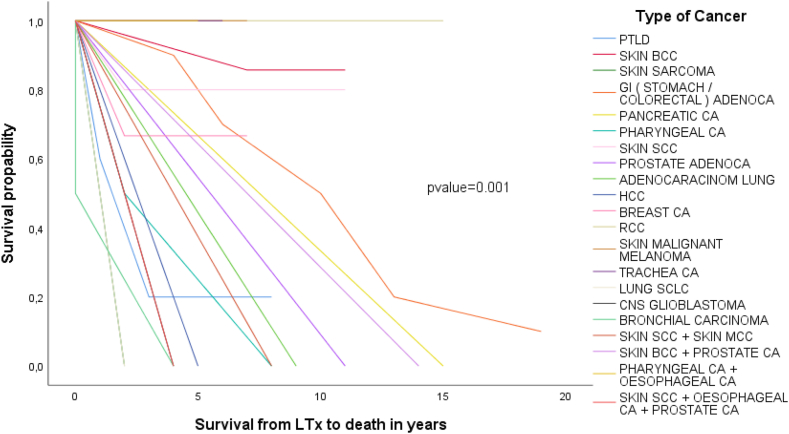
Kaplan-Meier survival by 20 years post-LuTx by type of the malignancy.

Survival rates in the n = 55 recipients who developed malignancy was 98.2% (n = 54) at 1 year, (n = 36) 65.5% at 5-years, and (n = 13) 23.6% at 10-years after LTx respectively. One recipient died 3 months after LTx due to acute lung rejection and lung cancer but a detailed histological and clinical report is missing. Average survival after malignancy onset was of 27 (range 1–104) months. N = 21/55 (38.2%) died during the first year after malignancy occurrence. All recipients who did not survive the malignancy died by the 8th year after onset. Moreover, the type of malignancy related significantly to the post-LuTx-survival and to elapse time ((p-values <0.05). In detail, pancreas showed 180, three synchronous malignancies 168, colon 135.42 ± 53.53, genital 132, stomach 66.00 ± 8.48, liver 60, pharynx 57 ± 55.15, skin 50.40 ± 36.39, CNS 48, lung 44.00 ± 46.73, MDS 27 ± 4.24, breast 24, and PTLD 12 months of post-LuTx survival respectively (p-value = 0.001). Recipients with skin malignancy had the longest mean post-LuTx survival time equal to 13 years. Kaplan-Meier of 20 years survival analysis after LuTx are presented in Fig. 1, Fig. 2.

**Fig. 3:**
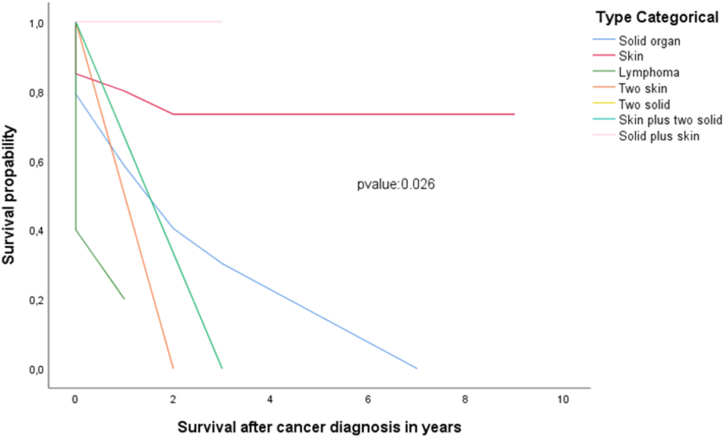
Kaplan-Meier survival by 10 years post-malignancy diagnosis by location of the malignancy.

**Fig. 4:**
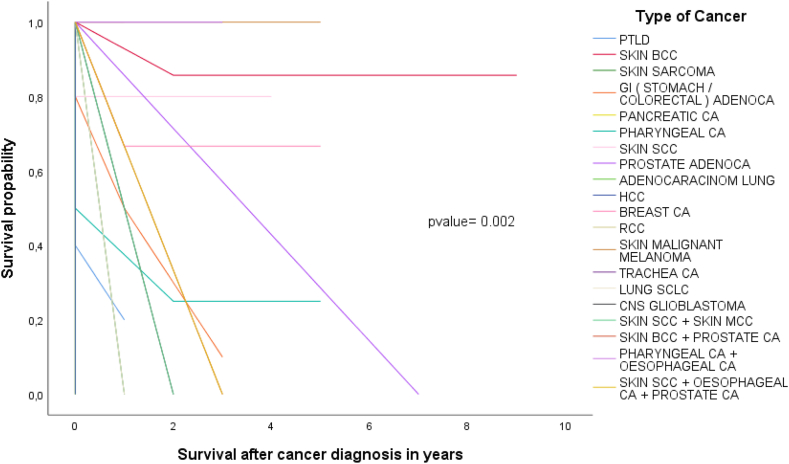
Kaplan-Meier survival by 10 years post-malignancy diagnosis by type of the malignancy.

When malignancies were divided based on their skin, solid-organ, and lymphatic origin, their origin was significantly related to mortality [X^2^(5.55)13.669, p-value: 0.018]. Lymphatic Malignancies showed significantly higher mortality rates of 80%, of solid organs 73.1%, and those of skin 25% respectively. Mortality ratio was 19/26 (73.1%) for solid organs, (of these, 90% was of GI adenocarcinoma) 5/20 (25%) for skin, 4/5 (80%) for PTLD, 1/2 (50%) for synchronous primary skin, and solid malignancies, 1/1 (100%) for two synchronous primary solid organs malignancies and 1/1 (100%) for three (2 solid, 1 skin) synchronous primary malignancies.

When malignancies were divided based on their type, mortality rates varied greatly (Table 3). All patients who developed lung adenocarcinoma, hepatic, pancreatic, other unspecified bronchial carcinomas, glioblastoma, sarcoma, combined SCC and MCC, combined pharyngeal SCC and esophageal carcinoma, as well as combined SCC, prostate and GI adenocarcinoma did not survive to the time of the study, representing a 100% mortality.

A total of 9/31 (29.03% of all MID) recipients with GI adenocarcinoma died in the study, n = 8 (25.8% of MID) died of the malignancy per se, and n = 1 (3.2%) of them died of a non-malignancy abdominal complication. 4/31 (12.9% of MID) recipients with PTLD died in the study, and all of them had malignancy as the cause of death, 3/31 recipients (9.7% of MID) with platen-epithelial carcinoma of the pharynx died, all of them had malignancy as the cause of death. BCC carcinoma was not determined to cause death in any case. One recipient (n = 1/31, 3.2%) developed glioblastoma and died of the non-malignancy caused by abdominal complication and sepsis. The recipient (n = 1/31, 3.2%) who developed SCLC had a combined cause of death, of malignancy complicated with acute rejection and sepsis. As presented in Table 5, there were significant differences between the malignancies of different origin and time intervals of LuTx-death (p = 0.019), and LuTx -malignancy onset (p-value: 0.003).

**Table 4:** Mann-Whitney Test, Mortality and continuous variables of Lung recipients with malignant disease.

Variables	Dead Median (IQR)	Alive Median (IQR)	p-value
Age at LuTx	54.00 (46.00–58.00)	57.00 (51.00–59.75)	0.168
follow-up after LuTx (years)	5.00 (2.00–10.25)	7.00 (5.00–8.75)	0.196
Survival from LuTx to death/time of the study (months)	60.00 (24.00–120.00)	84.00 (60.00–105.00)	0.141
Age at the time of malignancy diagnosis (years)	59.50 (49.50–66.00)	60.00 (58.00–65.12)	0.461
Time interval from LuTx to malignancy onset (months)	53.50 (18.50–100.50)	39.00 (16.75–68.00)	0.569
Survival after malignancy diagnosis (months)	9.00 (3.25–24.00)	37.00 (16.50–61.00)	0.000

**Table 5:** Malignancy origin and other parameters.

*Malignancy origin*	Survival from LuTx to death (months)	Age by malignancy onset (years)	Time from LuTx to malignancy onset (months)	Survival after malignancy onset (months)
***CNS***	48.00	*	28.00	26.00
***Skin***	75.86 ± 32.93	57.72 ± 7.43	45.22 ± 30.62	33.77 ± 28.91
***Breast***	52.00 ± 30.19	57.16 ± 6.78	16.33 ± 6.65	39.00 ± 27.18
***Colon***	147.00 ± 59.39	57.62 ± 11.99	129.62 ± 54.38	21.75 ± 14.33
***Genital***	92.00 ± 42.14	59.50 ± 7.36	40.66 ± 30.73	58.00 ± 35.08
***Liver***	92.00 ± 42.14	59.50 ± 7.36	40.66 ± 30.73	58.00 ± 35.08
***Stomach***	66.00 ± 8.48	66.00	60.50 ± 2.12	5.00 ± 5.65
***Lung***	44.00 ± 46.73	46.00 ± 31.11	53.00 ± 60.81	6.00 ± 4.24
***PTLD***	12.00	47.00 ± 3.53	8.50 ± 2.12	8.50 ± 10.60
***Pharynx***	66.00 ± 42.00	57.33 ± 7.50	34.66 ± 33.84	27.33 ± 31.06
***Pancreas***	180.00	61.00	180.00	1.00
*p-value*	0.001	0.317	0.003	0.450

*missing detailed data.

**Fig. 5:**
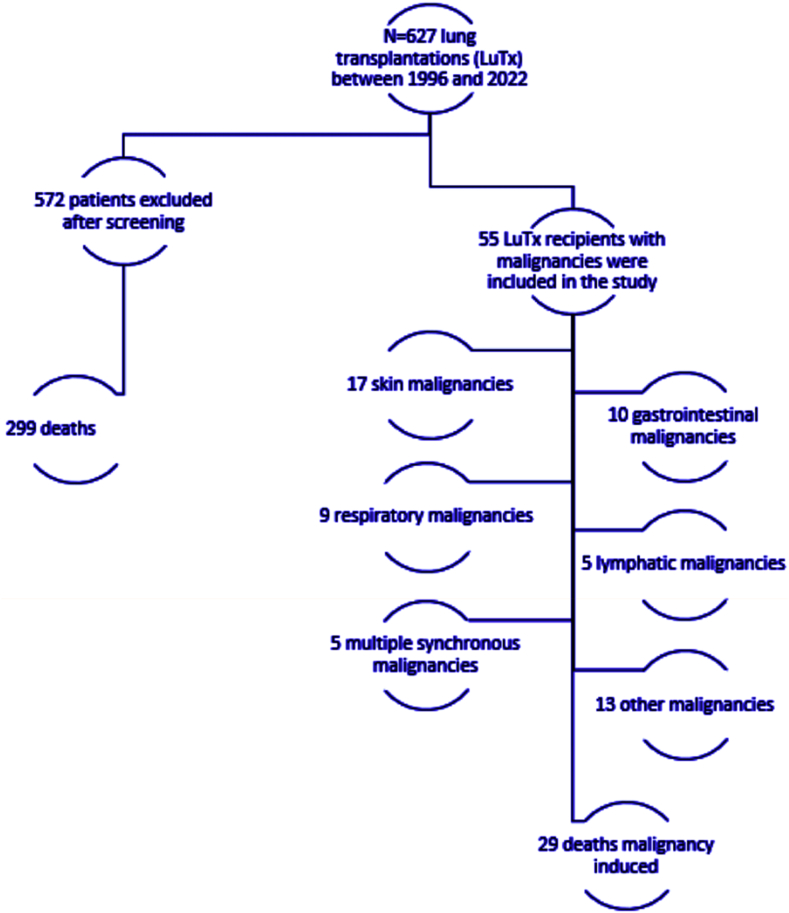
Flowchart showing patient selection and incidence of post-LuTx malignancies.

There was no significant difference between the different types and the age at malignancy occurrence (p-value = 0.317), or the survival after malignancy occurrence (p-value: 0.450). Fig. 5, summarizes the patient selection, LuTx malignancies incidence and deaths of this cohort.

### 3.5 Malignancy as the cause of death and other factors

There was statistically significant dependence between type of malignancy and malignancy as the cause of death (X^2^(19.31):95.424, p-value: 0.002), the primary LuTx diagnosis (X2 (28.31):42.711, p-value: 0.037), as well as a specific age (X^2^(1.31):21.815, p-value: 0.040). Specifically, 60% (15/25) of all recipients with COPD as the primary diagnosis who also developed malignancy, died of malignancy, this reflects 51.7% (15/29) of all MID. Moreover, n = 17/29 (58%) of the MID occurred in the age group of 50–64. However, malignancy as cause of death was independent of gender [X^2^(1.31)5.711, p-value: 0.222] and the type of LuTx they received [X^2^(6.31) 0.317, p-value: 1.000]. Table 4 shows further information regarding differences between recipients that survived and those that died of post-LuTx malignancies.

## 4 Discussion

This study presents the prevalence, characteristics, effects on survival, and mortality of post- LuTx malignancies. To the best of our knowledge, this is the first single-center report of its kind in such a large cohort of german LuTx recipients. The patterns of post-LuTx malignancies risk ratio, incidence, and survival vary among geographic regions for the different types of malignancies and for the different groups of transplant recipients [21]. Berastagui et al., based on the International Transplant Registry of Spain with a cohort of 1353 LuTx recipients, showed a post-LuTx malignancy incidence of 9.7% (6.9%–13.5%) at 5 years and 22.6% (16.5%–30.6%) at 10 years, with the most common to be skin cancer, PTLD, and lung cancer [2]. Collett et al. [21], based on the UK Transplant Registry and data from cancer registries in England, Scotland, and Wales showed non-melanoma skin cancer to be the commonest after thoracic transplantation. A study from Czech reports the lung cancer to be the most prelevant tumor and the second most frequent to be non-melanoma skin tumors after LuTx [22]. A prospective Swedish study of 331 LuTx patients with underlying end-stage COPD demonstrated the most common non-skin cancers to be lung cancer, colorectal cancer, and non-Hodgkin Lymphoma [23]. Schettini-Soares et al. from Brazil reported a post-LuTx incidence of 10.3% and found that the most common post-LuTx malignancy was non-melanoma skin cancer, prostate cancer, and PTLD [24]. Otani et al. from Japan, showed an incidence of 6.9% for all post-LuTx malignancies, with PTLD to be the most common [25].

The commonest primary diagnosis in our cohort were COPD and LF. This correlates to bibliography regarding the leading primary LuTx-indications [11]. The majority (96,4%) of our recipients received a DLuTx. This is in accordance with the ISHLT report 2019, showing that DLuTx accounts for 81% of all LuTx [11]. Furthermore, the median recipient age has increased from 50 to 57 years (p < 0.0001), and the proportion of male gender has also increased from 52% to 58% in the recent era (p < 0.0001) [26]. In our cohort, most of the recipients were males in the group age of 50–64 years.

In our study, the incidence of post-LuTx malignancies was 9.4% and confirms this of previous reports [2]. Recipient age and gender did not play a significant role either in the malignancy incidence or in the survival. This is a change from prior studies, in which the age and sex of lung recipients played a significant role in malignancies’ occurrence [2,10,27].

In our cohort, the prevalence ranking of the three commonest malignancies was skin > colorectal > PTLD. When dividing the malignancies in skin and non-skin, the total number of non-skin was higher (56% of total). Further, of all non-skin malignancies, the total of solid organ malignancies was the highest (47,5% of total). Thus, when categorizing the malignancies based on the affected organs, the most affected organ was skin. In our experience, and as shown in bibliography, non-melanoma post-LuTx malignancies are the commonest [10]. Non-melanoma skin malignancies occur earlier, more aggressively and show higher rates of metastasis and mortality in lung recipients than in the general population. Moreover, lung recipients have a 4–10 times higher risk for BCC, with an incidence of approximately 11.4% for BCC and 26.5% for SCC [28,29]. This is in agreement with our results demonstrating that 34% of all (n = 20/59, including cases of single and combined primary) malignancies were skin malignancies and of these, n = 19/21, 90.5% were non-melanoma malignancies. The commonest type was BCC (18.6% of all malignancies, 55% of skin, 58% of non-melanoma malignancies, 1.75% incidence in the cohort) showing a higher incidence than SCC (11,9% of malignancies, 35% of skin, 37% of non-melanoma, 1% Incidence in cohort). The total of BCC and SCC was 30.5% of all malignancies, and 90% of all non-melanoma skin malignancies. Merkelcell carcinoma is a rare, aggressive malignancy developing 24 times more in lung recipients [30]. We report one case of combined MCC and SCC carcinoma.

PTLD represents a heterogeneous lymphatic disease complex and although it was one of the commonest malignancies in lung recipients [8,31], its current incidence is 3–9% and tends to further decline thanks to the modern immunosuppression strategies [32]. In our study, PTLD incidence was 0.8% in the entire cohort, accounting for 8.5% of all malignancies, and affected 9% of recipients complicated with malignancies. This is lower than PTLD rates in previous studies [2] and may reflect differences in immunosuppression and EBV epidemiological patterns. Moreover, in our study, the PTLD had the fastest onset of all, mostly occurring during the first post-LuTx year, and showed high mortality rates (80% of PTLD patients died in our cohort) in agreement with previous reports [33].

The risk for lung malignancies in transplant recipients is 5 to 25-fold higher compared to the general population, and the highest among solid organ recipients, with an incidence of 1–9% post-LuTx [7,9,[34], [35], [36], [37]] much higher than that of the general population [38]. In our study, four recipients were diagnosed with lung cancer, consequently showing a 0.64% lung cancer incidence in the entire cohort and 6.8% of all post-LuTx malignancies, which is slightly lower than prior studies [39].

Although all recipients not surviving the malignancy died during the first 8 years after its onset, we observed a wide range of survival after onset among the different malignancies. This may relate to a more aggressive behavior of some malignancies in transplant populations [40]. Interestingly, the survival after onset also differed among the most lethal malignancies in our cohort. For example, pancreatic carcinoma had the latest onset, caused 100% mortality, and showed the shortest survival after diagnosis. This is due to its known aggressive behavior and often its late detection. Contrarily, among all malignancies with 100% mortality, glioblastoma showed the longest survival. Additionally, GI tract adenocarcinoma had the same incidence as BCC skin but caused 9-fold higher mortality (90% vs 10% of patients died respectively). The majority of MID was attributed to GI adenocarcinoma, and PTLD. On the other hand, melanoma, tracheal SCC, SCLC, and RCC showed 0.0% mortality at this point. This is in agreement with studies that prove significantly stronger associations with cancer-specific mortality for cancers with a typically better prognosis compared to malignancies that are more lethal for non-transplant populations [40].

A study limitation is its single-center retrospective nature. Furthermore, we lacked of data on the exact occurrence time and histological type of malignancy in two recipients. Additionally, some cases of malignancies may always be underdiagnosed, misdiagnosed, or lost in follow-up. It should be taken into account that some lung diseases per se contribute to the generation of malignancies (for example Cystic Fibrosis) [6]. Moreover, 5 years mortality is independently related to the primary diagnosis of cystic fibrosis [26]. Furthermore, the ReLuTx procedure is associated with lower survival and higher risk of both overall [41] and malignancy-specific mortality [40]. Additionally, further epidemiological, demographic, and other factors such as higher sun, radiation exposure, environmental mixtures, and chemical carcinogens between different regions, dietary habits, racial disparities, intrinsic genomic differences, and a more vulnerable lymphatic system to immunosuppression can influence the prevalence and outcome of these malignancies [19,42,43]. Finally, we cover a wide interval of 26 years. Consequently, the year-by-year variability in malignancies' incidence rates also reflects the combination of changing malignancy behavior patterns associated with varying cancer risk, new medical strategies, and cancer screening trends over time. The strengths of this report are the cohort's size and the presentation of the site- and type-specified incidence and mortality.

## 5 Conclusion

In conclusion, we proved an increased incidence of malignancies in a large LuTx cohort. Skin, colorectal, and PTLD are the most relevant with significant mortality. Our results provide a better understanding of malignancies after LuTx, underline the value of strict screening and high suspicion for the early detection of these, as well as the mandatory significance of a multidisciplinary approach to obtain satisfactory results in terms of improved quality of life and long-term survival. Thanks to the continuous progress in the LuTx field, the long-term survival of LuTx recipients is steadily improving. However, the more the post-transplant survival expands, the higher the malignancies-related risk, incidence, morbidity, and mortality of this aging population to expect. Future studies are needed to examine the donor and recipient risk factors, and the improvement of prevention, detection, and treatment to further handle the post-transplant obstacle of malignancies and reach better long-term results.

## Funding sources

None.

## Data availability statement

Data included in article/supp. Material/referenced in article.

## CRediT authorship contribution statement

**Konstantina Spetsotaki:** Conceptualization, Formal analysis, Investigation, Methodology, Project administration, Software, Validation, Visualization, Writing – original draft, Writing – review & editing. **Achim Koch:** Data curation, Funding acquisition. **Christian Taube:** Data curation, Formal analysis, Funding acquisition, Investigation. **Dirk Theegarten:** Funding acquisition, Resources, Software, Supervision. **Markus Kamler:** Funding acquisition, Resources, Software, Supervision. **Nikolaus Pizanis:** Resources, Software, Supervision, Data curation, Formal analysis.

## Declaration of competing interest

The authors declare that they have no known competing financial interests or personal relationships that could have appeared to influence the work reported in this paper.
